# Supplementary material for: Principles of cellular resource allocation revealed by condition-dependent proteome profiling
Source: eLife. 2017 Aug 31;6:e28034. doi: 10.7554/eLife.28034 (PMC5578734; doi:10.7554/eLife.28034)
Supplement: Transparent reporting form [file elife-28034-transrepform.docx]

***eLife’s* transparent reporting form**

We encourage authors to provide detailed information *within their submission* to facilitate the interpretation and replication of experiments. If you have any questions, please contact us: [editorial@elifesciences.org](mailto:editorial@elifesciences.org).

**Sample-size estimation**

- You should state whether an appropriate sample size was computed when the study was being designed
- You should state the statistical method of sample size computation and any required assumptions
- If no explicit power analysis was used, you should describe how you decided what sample (replicate) size (number) to use

Please outline where this information can be found within the submission (e.g., page numbers or figure legends), or explain why this information doesn’t apply to your submission:

This paper is based on omics data and was performed in biological repeats or temporal dynamics in order to increase the robustness of our findings.

**Replicates**

- You should report how often each experiment was performed
- You should include a definition of biological versus technical replication
- The data obtained should be provided and sufficient information should be provided to indicate the number of independent biological and/or technical replicates
- If you encountered any outliers, you should describe how these were handled
- Criteria for exclusion/inclusion of data should be clearly stated
- High-throughput sequence data should be uploaded before submission, with a private link for reviewers provided (these are available from both GEO and ArrayExpress)

Please outline where this information can be found within the submission (e.g., page numbers or figure legends), or explain why this information doesn’t apply to your submission:

Unless indicated in the figure caption, all replicates reporting can be found at the appropriate material and methods sections.

We excluded two strains in figure 2B, upper panel yellow circles, because the microarray data and the RNAseq were poorly correlated in the two source papers.

**Statistical reporting**

- Statistical analysis methods should be described and justified
- Raw data should be presented in figures whenever informative to do so (typically when N per group is less than 10)
- For each experiment, you should identify the statistical tests used, exact values of N, definitions of center, methods of multiple test correction, and dispersion and precision measures (e.g., mean, median, SD, SEM, confidence intervals; and, for the major substantive results, a measure of effect size (e.g., Pearson's r, Cohen's d)
- Report exact p-values wherever possible alongside the summary statistics and 95% confidence intervals. These should be reported for all key questions and not only when the p-value is less than 0.05.

Unless indicated in the figure and in the figure caption, all relevant statistical reporting can be found at the appropriate material and methods sections.

Please outline where this information can be found within the submission (e.g., page numbers or figure legends), or explain why this information doesn’t apply to your submission:

(For large datasets, or papers with a very large number of statistical tests, you may upload a single table file with tests, Ns, etc., with reference to page numbers in the manuscript.)

**Additional data files (“source data”)**

- We encourage you to upload relevant additional data files, such as numerical data that are represented as a graph in a figure, or as a summary table
- Where provided, these should be in the most useful format, and they can be uploaded as “Source data” files linked to a main figure or table
- Include model definition files including the full list of parameters used
- Include code used for data analysis (e.g., R, MatLab)
- Avoid stating that data files are “available upon request”

Please indicate the figures or tables for which source data files have been provided:

Source files were uploaded for the transcriptomic figures and are available via the SRA service, accession # **SRP107059**.

External datasets were obtained from (see also Materials and Methods):

•	Gasch, A.P., Spellman, P.T., Kao, C.M., Carmel-Harel, O., Eisen, M.B., Storz, G., Botstein, D., and Brown, P.O. (2000). Genomic Expression Programs in the Response of Yeast Cells to Environmental Changes. Mol. Biol. Cell 11, 4241–4257.

•	Kemmeren, P., Sameith, K., Van De Pasch, L.A.L., Benschop, J.J., Lenstra, T.L., Margaritis, T., O’Duibhir, E., Apweiler, E., Van Wageningen, S., Ko, C.W., et al. (2014). Large-scale genetic perturbations reveal regulatory networks and an abundance of gene-specific repressors. Cell 157, 740–752.

•	Murphy, J.P., Stepanova, E., Everley, R.A., Paulo, J.A., and Gygi, S.P. (2015). Comprehensive Temporal Protein Dynamics during the Diauxic Shift in Saccharomyces cerevisiae. Mol. Cell. Proteomics 14, 2454–2465.

•	O’Duibhir, E., Lijnzaad, P., Benschop, J.J., Lenstra, T.L., van Leenen, D., Groot Koerkamp, M.J., Margaritis, T., Brok, M.O., Kemmeren, P., and Holstege, F.C. (2014). Cell cycle population effects in perturbation studies. Mol. Syst. Biol. 10 VN-r, 732.

•	Paulo, J.A., O’Connell, J.D., Gaun, A., and Gygi, S.P. (2015). Proteome-wide quantitative multiplexed profiling of protein expression: carbon-source dependency in Saccharomyces cerevisiae. Mol. Biol. Cell 26, 4063–4074.

•	Paulo, J.A., O’Connell, J.D., Everley, R.A., O’Brien, J., Gygi, M.A., and Gygi, S.P. (2016). Quantitative mass spectrometry-based multiplexing compares the abundance of 5000 S. cerevisiae proteins across 10 carbon sources. J. Proteomics 148, 85–93.

•	Tamari, Z., Rosin, D., Voichek, Y., and Barkai, N. (2014). Coordination of gene expression and growth-rate in natural populations of budding yeast. PLoS One 9, e88801.

•	Tamari, Z., Yona, A.H., Pilpel, Y., and Barkai, N. (2016). Rapid evolutionary adaptation to growth on an “unfamiliar” carbon source. BMC Genomics 17, 674.

Wang, M., Weiss, M., Simonovic, M., Haertinger, G., Schrimpf, S.P., Hengartner, M.O., and von Mering, C. (2012). PaxDb, a database of protein abundance averages across all three domains of life. Mol. Cell. Proteomics 11, 492–500.

Gene groups’ names are indicated in Supplementary table 1&2.
